# Biomarker Acquisition and Quality Control for Multi-Site Studies: The Autism Biomarkers Consortium for Clinical Trials

**DOI:** 10.3389/fnint.2019.00071

**Published:** 2020-02-07

**Authors:** Sara Jane Webb, Frederick Shic, Michael Murias, Catherine A. Sugar, Adam J. Naples, Erin Barney, Heather Borland, Gerhard Hellemann, Scott Johnson, Minah Kim, April R. Levin, Maura Sabatos-DeVito, Megha Santhosh, Damla Senturk, James Dziura, Raphael A. Bernier, Katarzyna Chawarska, Geraldine Dawson, Susan Faja, Shafali Jeste, James McPartland, the Autism Biomarkers Consortium for Clinical Trials, Adham Atyabi

**Affiliations:** ^1^Center on Child Health, Behavior, and Development, Seattle Children’s Research Institute, Seattle, WA, United States; ^2^Department of Psychiatry and Behavioral Sciences, University of Washington School of Medicine, Seattle, WA, United States; ^3^Department of Pediatrics, University of Washington School of Medicine, Seattle, WA, United States; ^4^Duke Center for Autism and Brain Development, Duke University, Durham, NC, United States; ^5^Department of Biostatistics, University of California, Los Angeles, Los Angeles, CA, United States; ^6^Department of Psychiatry and Biobehavioral Sciences, University of California, Los Angeles, Los Angeles, CA, United States; ^7^Department of Statistics, University of California, Los Angeles, Los Angeles, CA, United States; ^8^Yale Child Study Center, Yale University, New Haven, CT, United States; ^9^Department of Neurology, Boston Children’s Hospital, Boston, MA, United States; ^10^Harvard Medical School, Harvard University, Boston, MA, United States; ^11^Center on Human Development and Disability, University of Washington, Seattle, WA, United States; ^12^Department of Pediatrics, Boston Children’s Hospital, Boston, MA, United States; ^13^Department of Neurology, University of California, Los Angeles, Los Angeles, CA, United States

**Keywords:** autism spectrum disorder, biomarkers, clinical trial methods, guidelines, EEG, eye tracking, video tracking

## Abstract

The objective of the Autism Biomarkers Consortium for Clinical Trials (ABC-CT) is to evaluate a set of lab-based behavioral video tracking (VT), electroencephalography (EEG), and eye tracking (ET) measures for use in clinical trials with children with autism spectrum disorder (ASD). Within the larger organizational structure of the ABC-CT, the Data Acquisition and Analytic Core (DAAC) oversees the standardization of VT, EEG, and ET data acquisition, data processing, and data analysis. This includes designing and documenting data acquisition and analytic protocols and manuals; facilitating site training in acquisition; data acquisition quality control (QC); derivation and validation of dependent variables (DVs); and analytic deliverables including preparation of data for submission to the National Database for Autism Research (NDAR). To oversee consistent application of scientific standards and methodological rigor for data acquisition, processing, and analytics, we developed standard operating procedures that reflect the logistical needs of multi-site research, and the need for well-articulated, transparent processes that can be implemented in future clinical trials. This report details the methodology of the ABC-CT related to acquisition and QC in our Feasibility and Main Study phases. Based on our acquisition metrics from a preplanned interim analysis, we report high levels of acquisition success utilizing VT, EEG, and ET experiments in a relatively large sample of children with ASD and typical development (TD), with data acquired across multiple sites and use of a manualized training and acquisition protocol.

## Introduction

To develop more targeted diagnostic and treatment methods to improve outcomes in autism spectrum disorder (ASD) (Loth et al., 2016), the scientific field must address the current lack of reliable and sensitive objective measures that inform treatment target engagement or subgroup identification (Jeste et al., 2015; McPartland, 2017; Sahin et al., 2018; Ewen et al., 2019). The Autism Biomarkers Consortium for Clinical Trials (ABC-CT^1^) was created to advance biomarker validation for eventual use in clinical trials for children with ASD with a number of potential contexts of use, including reduction of heterogeneity of samples via stratification, potential for indication of early efficacy or demonstration of target engagement, and outcome measurement (McPartland, 2016). The ABC-CT is a response to the RFA-MH-15-800 U19 Consortium on Biomarker and Outcome Measures of Social Impairment for use in Clinical Trials in ASD. To this end, the ABC-CT consortium (McPartland et al., 2019) is evaluating behavioral video tracking (VT), electroencephalography (EEG), and eye tracking (ET) as indices of *social communication* for potential use in ASD clinical trials—as social communication is one of the core targets for pharmacological and behavioral interventions (e.g., Lerner et al., 2012; Anagnostou, 2018).

In this report, we articulate the standard operating protocols developed by the ABC-CT Data Acquisition and Analytic Core (DAAC) related to: (1) the design and implementation of multi-site experimental protocols and (2) the quality control (QC) processes related to rigorous, scientifically valid, and replicable procedures used for data acquisition. Unlike a traditional theoretical or empirical paper describing clinical findings (which will be described in a companion manuscript), we focus on methods of acquisition and the rationale for these choices—addressing the question “can a biomarker be measured accurately?” (Institute of Medicine (US) Committee on Qualification of Biomarkers and Surrogate Endpoints in Chronic Disease, 2010; Amur et al., 2015). To oversee consistent application of scientific standards and methodological rigor for data acquisition, processing, and analytics, we developed standards of work that reflect the logistics of multi-site research, and the need for well-articulated, transparent processes that can be implemented by the scientific community in future clinical trials of children with ASD and other neurodevelopmental disorders. As these processes often reflect the internal workings of a study or laboratory, but are critical for replication and/or use in future clinical trials, full transparency of these processes is critical when considering the potential for broad implementation.

## Protocol

The ABC-CT study was conducted in two phases: a Feasibility phase and a Main study phase. The Feasibility Study (see section “Feasibility Study” for details) was conducted to address whether or not the methods could be successfully implemented for the participant group across the five sites consistently in a small sample (*n* = 50, 50% ASD). After review of results from the Feasibility Study, the Main Study battery was developed with a goal of 275 participants [*n* = 200 ASD; *n* = 75 typical development (TD); aged 6–11 years], each observed at three timepoints (Time 1 = baseline, Time 2 = 6 weeks, Time 3 = 6 months) (and as specified in the RFA-MH-15-800). Data were evaluated at the interim point in the Main Study, at which approximately 50% of participants had been enrolled and completed the first and second timepoint. (Sample characteristics from the Main Study Interim Sample are presented in Supplementary Material).

Four principles guided the work of the ABC-CT across both phases: First, the study was conducted in partnership between the scientific key personnel and the NIH scientific and program officers, the FNIH Biomarkers Consortium, and an external advisory board. Second, all work was performed in accordance with Good Clinical Practice regulatory standards (FDA, 2019). Third, the data were acquired by clinical sites separate from both data management and data analytic teams. Fourth, the domains of assessment (see section “Domains”) included clinical characterization of the participants (both ASD and TD), automated behavioral assessments, EEG, and ET.

### Domains

#### Clinical Characterization

The sample of participants was characterized using autism diagnostic standardized measures, including the Autism Diagnostic Observation Schedule, 2nd Edition (Lord et al., 2012) and the Autism Diagnostic Interview – Revised (Rutter et al., 2003) (ADI-R). Participant behaviors were quantified in the following domains: Social communication; verbal and non-verbal ability; physical, medical, and psychological conditions; and psychotropic medications. All information was collected from both the participants with ASD and a TD control group (see Supplementary Material for the interim sample characteristics).

To allow for counterbalancing of the methods and experiments, at screening, participants were stratified based on variables that could be assessed by phone to include group (ASD/TD), biological sex (male/female), age (split at 8 years 6 months), and functioning (ASD only). Of note, pre-visit functioning for the Feasibility Study was identified based on response to the ADI-R question assessing functional language (Rutter et al., 2003); at Main Study, functioning was split based on a report of a full scale IQ above or below 80. These factors were used to create four stratification groups, which then directed the counterbalancing protocol. For Feasibility, this included method order (Table 1) and experiment order (Tables 2, 3); for Main Study, method order was fixed (Table 1 “Main Study Order”) but experimental order was randomized within method (Tables 2, 3).

**TABLE 1:** Acquisition methodology protocol order for Feasibility and Main Study.

	Feasibility Order-A	Feasibility Order-B	Feasibility Order-C	Feasibility Order-D	Main Study Order
Day 1	Behavior	Behavior	Behavior	Behavior	Behavior
	VT	VT	VT	VT	VT
	ET	ET	EEG	EEG	ET
	EEG	EEG	ET	ET	
Day 2	Behavior	Behavior	Behavior	Behavior	EEG
	ET	EEG	ET	EEG	ET
	EEG	ET	EEG	ET	

*Key: VT = video tracking, ET = eye tracking.*

**TABLE 2:** Acquisition experiment Order-A within EEG for Feasibility and Main Study.

	Day 1	Day 2
EEG Feasibility	Set 1	Set 2
	1. Resting eyes open	1. Resting eyes open
	2. EU-AIMS faces	2. Biomotion
	3. VEP	3. Emotion faces
		4. Social/non-social dynamic
EEG Main Study	No day 1 EEG	Day 2
		1. Resting eyes open
		2. ABC-CT faces
		3. VEP
		4. Biomotion

**TABLE 3:** Acquisition experiment order (A) within ET for Feasibility and Main Study for day 1 (left) and day 2 (right).

ET Feasibility	1. Pupillary light reflex 2. Spontaneous social orienting 3. Pupillary light reflex 4. Gap overlap 5. Pupillary light reflex 6. Gap overlap 7. Biological motion preference 8. Pupillary light reflex 9. Biological motion preference 10. Dynamic scenes 11. Pupillary light reflex 12. Social interactive 13. Pupillary light reflex 14. Social interactive 15. Pupillary light reflex 16. Activity monitoring 17. Pupillary light reflex 18. Activity monitoring 19. Pupillary light reflex	1. Pupillary light reflex 2. Dynamic scenes 3. Pupillary light reflex 4. Social interactive 5. Pupillary light reflex 6. Social interactive 7. Pupillary light reflex 8. Activity monitoring 9. Pupillary light reflex 10. Activity monitoring 11. Pupillary light reflex 12. Visual search/static scenes 13. Pupillary light reflex 14. Visual search/static scenes 15. Biological motion preference 16. Pupillary light reflex 17. Biological motion preference 18. Gap overlap 19. Pupillary light reflex 20. Gap overlap
ET Main Study	1. Pupillary light reflex 2. Activity monitoring 3. Pupillary light reflex 4. Activity monitoring 5. Pupillary light reflex 6. Biological motion preference 7. Pupillary light reflex 8. Biological motion preference 9. Pupillary light reflex 10. Social interactive 11. Pupillary light reflex 12. Social interactive 13. Pupillary light reflex 14. Visual search/static scenes 15. Pupillary light reflex 16. Visual search/static scenes 17. Pupillary light reflex	1. Pupillary light reflex 2. Social interactive 3. Pupillary light reflex 4. Social interactive 5. Pupillary light reflex 6. Visual search/static scenes 7. Pupillary light reflex 8. Visual search/static scenes 9. Pupillary light reflex 10. Activity monitoring 11. Pupillary light reflex 12. Activity monitoring 13. Pupillary light reflex 14. Biological motion preference 15. Pupillary light reflex 16. Biological motion preference 17. Pupillary light reflex
		

#### Behavioral Video Tracking of Child Behavior

The parent–child context is critical to children’s development and is often the target of early intervention models for children with ASD (e.g., Dawson et al., 2010; Kasari et al., 2010; Estes et al., 2014). Currently, it is standard practice to manually code children’s behaviors in the context of child play tasks. Such work is labor intensive, time consuming, prone to human error and subjectivity, and thus infeasible for large clinical trials. As a goal of the ABC-CT is to develop objective, reliable measures of social behavior that do not rely on parent report or clinical judgment, we implemented a behavioral protocol and post-acquisition automatic quantification of child motion and location via VT with the Noldus EthoVision XT (EVXT) 11.5 software (Sabatos-DeVito et al., 2019). We implemented EVXT as a potential objective, standardized, quantitative, and scalable measure of social approach in ASD in the context of a parent–child interaction (Cohen et al., 2014).

**FIGURE 1:**
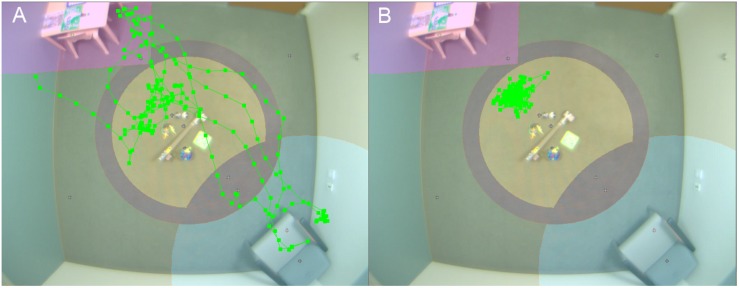
Video tracking of child physical movement. Room setup for PCFP with overlay of video tracking of movement of child 1 **(A)** and 2 **(B)**.

For the ABC-CT, VT was used to provide automated measures of voluntary physical approach-withdrawal toward a social partner (the parent) in the context of a parent–child free play (PCFP) session (see Figure 1 for an example of child physical movement). In both the Feasibility and Main Study, the PCFP included a standard room setup (furniture, module-based toy kit, and parent placement) that allowed for the child to move about the environment and engage in solitary, interactive, or social play. During the PCFP, the parent sat in a chair, readily available for interaction if and when approached by the child, while the child freely explored the room and available toys. The location of the toys and parent in relation to the ceiling-mounted Noldus camera allowed for tracking of child location, including time and frequency of interaction in various regions of interest (Sabatos-DeVito et al., 2018).

#### EEG

Scalp electrophysiological recordings are a non-invasive method of measuring the brain’s electrical activity. EEG does not require the participant to produce motor or verbal responses and can be collected from experimental paradigms requiring no overt response. The methodology can thus be used across the lifespan and with participants who have limited cognitive or communicative abilities. It also offers opportunities for translational research across species. Despite strong theoretical and methodological arguments for the use of EEG in understanding the neural correlates of autism (Jeste et al., 2015; Loth et al., 2017; McPartland, 2017), the practice of collecting, processing, and evaluating EEG data is complex, particularly when data acquisition involves children or those with developmental or cognitive disabilities. Descriptions of basic methodology can be found in a number of published texts and guidelines (Pivik et al., 1993; Picton et al., 2000) and specifically related to use in ASD (Webb et al., 2015).

In the ABC-CT, EEG acquisition included six paradigms addressing basic brain functioning as well as social ability and understanding. The six experiments in the Feasibility Study were reduced to four in the Main Study (Webb et al., 2018): (1) Resting EEG eyes open during calm viewing of digital videos (similar to screensavers). (2) Event-related responses to upright and inverted faces compared to upright houses, targeting early stage attention and perception of social information. [Note: in the Feasibility Study this paradigm was the same as that employed in the EU-AIMS protocol (Loth et al., 2017) but an additional object stimulus condition was included, which then was also implemented in phase 2 of the EU-Aims protocol. For the Main Study, the paradigm was altered to utilize a pre-stimulus fixation crosshair as in Webb et al. (2012), while the EU-AIMS version utilized a pre-stimulus object icon.] (3) Event-related responses to biological motion (“biomotion”), investigating the responses to coherent and scrambled point light animation of adult male walkers (Naples, Webb, et al., in development); and (4) visual evoked potentials (VEPs) elicited by an alternating black and white checkerboard (1 Hz) to assess functional integrity of the afferent visual pathway and basic visual processing (LeBlanc et al., 2015). The two experiments included in Feasibility but excluded from Main Study were: (5) event-related response to fear and neutral facial expressions (Dawson et al., 2004) and (6) EEG to social and non-social dynamic videos (Jones et al., 2015, 2016), which is included in the EU-AIMS battery.

#### Eye Tracking

Remote video oculographic ET uses a video of participant’s eyes to determine point of regard (POR) on a computer screen, with this video also often allowing for a measure of pupil diameter (Shic, 2013). This POR is considered a proxy for visual attention in practical, real-world situations and is associated both with the cognitive information processing of attended-to locations as well as the motivational process involved in selection of PORs (Kowler, 1990). Modern ET relies primarily upon video oculographic techniques which (as compared to other ET techniques, such as scleral coils) are non-invasive, highly tolerable, robust to movement, and can provide quantitative data on looking patterns at less than a degree of visual angle and with millisecond timing (Duchowski, 2007; Holmqvist et al., 2011; Shic, 2016). In autism research, the use of ET has matured, expanded, and seen widespread adoption over the past decade, and may offer a feasible early-efficacy biomarker in clinical trials (Dawson et al., 2012; Murias et al., 2017).

The ABC-CT ET included nine paradigms in Feasibility, reduced to five in the Main Study: (1) activity monitoring, which includes both static images and dynamic videos of two adult actors playing with children’s toys while gazing at each other or at their shared activity (Shic et al., 2011; Umbricht et al., 2017; Del Valle Rubido et al., 2018). (2) Biological motion preference, where two point-light displays are shown on either side of the screen, one displaying biological motion and one displaying a control condition of rotating or scrambled dots (Annaz et al., 2011; Umbricht et al., 2017; Del Valle Rubido et al., 2018). (3) Pupillary light reflex, in which a dark screen with a small fixation animation at the center is shown, then replaced briefly by a white screen, followed by the same dark screen with animation (Nyström et al., 2015). This task was interleaved between blocks of all other paradigms and came from the EU-AIMS protocol (Loth et al., 2017). (4) Social interactive, where children play with toys either together or separately with no sound (Chevallier et al., 2015) and (5) static scenes (SS), which included photographs of adults and children engaged in social activities. This task came from the EU-AIMS protocol. Included in Feasibility only were: (6) Dynamic naturalistic scenes, in which two, 4-min videos were shown (one on each day) that drew from clips of live-action movies (adapted from Rice et al., 2012). (7) Gap overlap, in which an animation was shown at the center of the screen and then a peripheral stimulus was displayed while the central stimulus was on screen (overlap condition), immediately after the central stimulus left the screen (baseline condition), or after the central stimulus left the screen (gap condition) (Elsabbagh et al., 2013a). (8) Spontaneous social orienting, which involved an actress speaking directly to the camera while conducting an activity and directing the participant’s attention to various toys (Chawarska et al., 2012) and (9) visual search, in which five images were displayed in a circle for the participant to free view (Sasson et al., 2011; Elsabbagh et al., 2013b). Note that visual search trials were interleaved with SS trials, as in the EU-AIMS protocol. To preserve the structure of the task, visual search trials were left in the Main Study protocol but were not prioritized in analysis.

### Equipment and General Experimental Structure

#### VT

Video data of interpersonal interaction were collected with an overhead color CCD IP camera with a wide-angle lens mounted in the center of the room and recorded using Noldus Media Recorder 3.0 software. A second side camera video with audio was added in the Main Study in order to enhance quality review of overhead recordings. The PCFP protocol was standardized including positioning of furniture, parent seating and behavior suggestions, and arrangement of child toys on the floor and on a table (Sabatos-DeVito et al., 2018). All sites utilized the same toys for the PCFP. Sites transferred collected data (overhead camera.avi file, side camera video recording) to a subject specific folder, compressed the folder, and then transferred the folder to a computer with internet access to the DCC database. The VT session log was entered via online data capture.

#### EEG

Each experiment was standardized (Borland et al., 2018) to start with a welcome screen, direction screen, general directions [“please sit still and watch the (insert stimulus)”] and start directions. Site specific seating distance modifications were used to ensure standard visual angle. All introduction screens included both text and audio. Experimenters were provided with additional sample language to support child understanding and compliance in regard to the method, order, and behavioral expectations.

Experiments were divided into blocks of about ∼2 min to facilitate participant attention, engagement, and compliance. Pauses or “rest time” occurred between blocks; the goal was to have block breaks of less than 2 min. Experiments were not allowed to be re-run or conducted out of order. The projected time for the EEG battery in the Main Study was 16.0 min, with no breaks; at Interim, the average actual run time (from time of start of first experiment, to end of last experiment) was 24.44 min (SD 7.1) suggesting greater use of break periods than seen in Feasibility.

**FIGURE 2:**
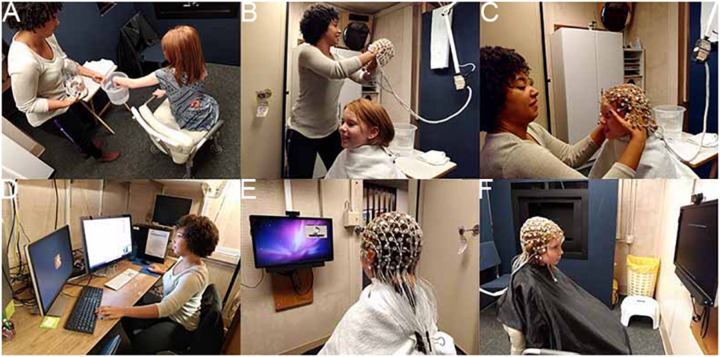
EEG session. **(A)** Participant exploring the EEG equipment; **(B)** preparing for the net; **(C)** net placement; **(D)** experimenter setup for monitoring experiment, data, and child attention; **(E)** child watching video while setup is finalized; and **(F)** child attending to instruction screen for experiment. Written consent was obtained from the adult experimenter and the parents of the child shown; the child provided assent.

As shown in Figures 2A–F, all sites had an EGI 128 channel EEG acquisition system, including both 300 and 400 amps, the 128 electrode EGI HydroCel Geodesic Sensor Nets (applied according to EGI standards), Logitech Z320 Speakers, Cedrus StimTracker (for visual presentation timing), and a monitor (Dell P2314H 23” resolution 1920 × 1080, Main Study) (Webb et al., 2018). Appropriate Net Station acquisition setups (1000 Hz sampling rate, 0.1–200 Hz filter, EGI MFF file format, onset recording of amplifier and impedance calibrations) were provided to each site. EPrime 2.0 was used for experimental control; a master experiment was created and then modifications from that master were made based on site differences. Experimental versions were tracked and acquisition files were verified to make sure the correct versions were implemented. Sites transferred collected data (EEG raw MFF file, session log, E-Prime data file) to a subject specific folder, compressed, and then transferred to the DCC database. The EEG session log was entered via online data capture.

#### Eye Tracking

**FIGURE 3:**
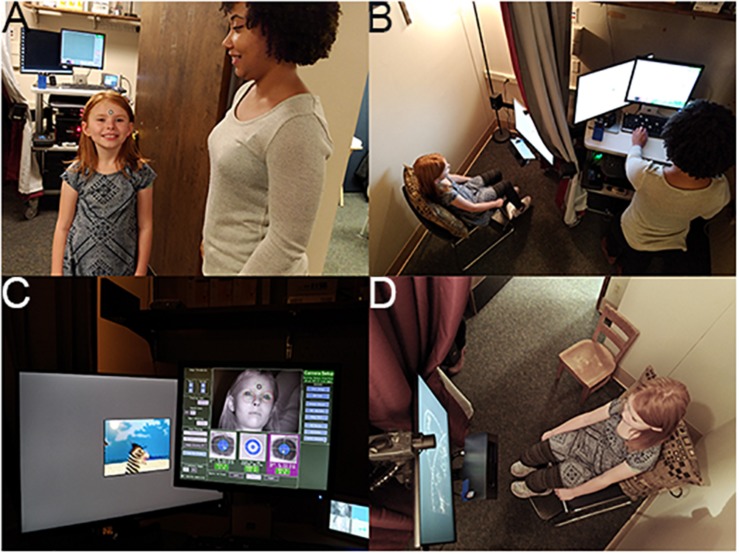
ET session. **(A)** Preparing to enter ET room and ET sticker placement; **(B)** overhead view of room with participant and experimenter; **(C)** experimenter setup for monitoring experiment, data, and child attention; and **(D)** child attention to experiment. Written consent was obtained from the adult experimenter and the parents of the child shown in the images; the child provided assent.

All sites collected ET data using SR Research Eyelink 1000 Plus binocular remote eye trackers at 500 Hz (in EDF file format) with 24″ Dell monitors for display (1920 × 1200 pixels) (Naples et al., 2018; Shic et al., 2018). Each participant was required to wear a target sticker on their forehead to allow the eye tracker to locate their eyes (Figure 3A). This sticker also allowed the computer to determine child-to-monitor distance. Participants were positioned at 650 mm from the ET camera at the start of each session. ET sessions had both an experimenter running the computers and a behavioral assistant sitting with the child to support them throughout the task if needed (Figure 3D). Experiments were presented in an integrated delivery system programmed in Neurobehavioral Stimulus Presentation version 18.1 that included an initial video to ease participant setup (including participant positioning and ET calibration), delivery of core experimental paradigms, embedded periodic ET calibration/validation routines, and the incorporation of routines to allow for experimenter-triggered breaks for behavioral management. Paradigm blocks lasted 1–4 min each and were interleaved to combat fatigue. The projected total time of the experiments was 15 min; average actual run time (including setup, calibration, to end of the experiment set) was 18.2 min (SD 2.3).

A Python script with a user interface was created to help sites compress the ET output files and video files that could then be transferred to the DCC database. The ET Run Logs were entered directly into the online database.

### Environment and Supports

Overall, the environment was to be free of distractions that might impede, interrupt, or alter performance differentially by child or site. Fixed characteristics of the sites’ data collection environments were taken into consideration in the design of the equipment and the analytic pipelines. These characteristics were tracked in the acquisition protocols and monitored during QC review. When alterations had to be made to the environment based on child characteristics that prohibited the use of the standard environment, this was noted as a protocol deviation on the methodology log. Note, we did have sites that changed room locations between Feasibility and Main Study—this occurred due to new spaces becoming available that better accommodated the participants and the equipment and was not done because of specific concerns with the rooms *per se*.

A number of physical (booster chairs, footstools, tables), social (social scripts or videos), and behavioral supports (visual schedules) were identified to facilitate individual child performance and were deemed not to interfere with the acquisition parameters or the psychological constructs being assessed. Questions about allowed supports were addressed during training and via the weekly coordinator call. These were noted in the logs, but not identified as a protocol deviation.

#### VT

Environmental effects were most obvious for the VT protocol in which room size and layout could not be made physically identical across sites. Detailed measurements of the room size, positioning of furniture, and PCFP items were created and each site was expected to maintain within-site standardization. The VT Manual of Operations (Sabatos-DeVito et al., 2018) details room variations, scaling procedures to standardize regions of interest, and their implications for abstraction of child positioning and movement in the EVXT software.

#### EEG

For EEG, because all site rooms had different lighting setups (type of lights, location in relation to participant/monitor), and concern about participant reaction to dark/dim lighting, we conducted all sessions in full room light. Due to room layout, the location of the behavioral assistant (who facilitated child compliance) differed by site but was standardized within a site. Sites were instructed to send monthly pictures of their lab setup to check compliance for room layout, including subject to monitor distance (for visual angle).

#### Eye Tracking

For ET, the equipment and acquisition setup was developed for installation on a cart or fixed location (see Figure 3). Behavioral assistants were allowed to be on either side of the child or behind on a case-by-case basis at each site. Ambient room lighting during sessions was monitored (via a light meter) and sites were instructed to keep the lighting dim but not completely dark. Before each session, sites were instructed to test the sound levels of their speakers using a test tone and an external sound meter. Sound was set at 65 dB. Sites were asked not to adjust sound or lighting during a session.

### Training

Training was provided by the DAAC via in-person site visits, online training, regular weekly phone calls, and written documents. All sites were at academic institutions, directed by PIs with extensive history of training in these methodologies, and thus, a decision was made to maintain staffing and basic training responsibilities with the site PI. The DAAC provided some general acquisition training, but focused primarily on the methodology for the ABC-CT protocol. For example, training in EGI net placement was done within the lab but training on the net placement scoring system was done by the DAAC trainer.

To be “certified” as collection staff, all personnel had to complete requirements at their institution (including human subjects training) and their PI’s current lab training protocol. Then the staff members received in-person training either with a DAAC acquisition lead or the onsite trainer, reviewed all written documents, and provided two to five protocol evaluation files for DAAC to review that demonstrated competence in acquiring valid data. New staff also had the first five sessions with participants (for each methodology) intensively reviewed by DAAC staff. Written feedback was provided to the staff member acquiring the data, site method lead, and PI. Feedback was provided during protocol evaluation training and during ongoing intensive review. This was manualized (Barney et al., 2018; Sabatos-DeVito et al., 2018; Santhosh et al., 2018). The DAAC conducted method-based meetings bi-weekly to review feedback reports and current site acquisition validity. Any significant issues identified by the DAAC were addressed via re-training at the Site. The DAAC acquisition lead attended the ABC-CT weekly coordinator call to answer questions and provide feedback related to acquisition. Site staff turnover was also monitored by the DAAC. Transition of the on-site trainer, more than 50% of acquisition staff, or request by PI triggered an on-site visit for training.

### Experimenter Roles and Interactions

The protocol “with child in room,” essentially the running of the experimental battery, was manualized so that each child experienced the same steps from the time upon entry into the lab space until departure. This included scripted language and actions. As one of our main analytic aims was to assess test–retest validity of our biomarker dependent variables (DVs), eliminating individual session variability was a key principle.

Because of variability in age of participants (6 years 0 month to 11 years 11 months) and functioning level (full scale IQ 60–150), pre-testing participant familiarization was not standardized and was left to individual decisions between the lead clinician, acquisition experimenter, and parent. The setup order as well as within methodology experimental order was not allowed to be altered, again because of concerns about how protocol modifications might impact test–retest validity. Thus, variability in changing order, or “moving” a method to a stand-alone session was not allowed. It is possible that utilizing a fixed acquisition protocol order lowered rates of acquisition for later tasks in children that might have been more fatigued by the battery or may have been able to succeed with more familiarization.

Correct identification of no data or poor data was deemed to be of high priority for sites during acquisition for two reasons: First, acquisition rates were critically important for sites to set subject flow and update enrollment targets. Second, site monitoring and feedback included establishing when no or poor data resulted from valid participant interactions (e.g., the session was run correctly but the child did not have the behavioral skills to comply with the method) versus quality of experimenter interactions (e.g., the experimenter made decisions that did not support collection of valid data).

Staff monitoring of child behavior for data validity occurred through two main methods: (1) Monitoring and coding behavior online during the experiment and (2) logging child behavior per method per experiment per block on a standard form. For monitoring online, ET and EEG experiments were coded such that non-attention or non-compliance could be recorded (via a keypress) in the raw data recording. This allowed for tracking of behavior moment-to-moment.

Acquisition staff utilized a methodology-specific session log, which included child characteristics (e.g., description of child for video-log identity matching, child head size), session characteristics (e.g., start time, child positioning, distance to monitor, staff location, parent presence), and method-specific details that might impact post-acquisition processing (e.g., EEG-net fit impacting signal acquisition; VT-presence of red in room interfering with person tracking; ET-ambient room light levels that could impact pupil size). For logging of general behaviors, several drafts of the logs were attempted, with the final reflecting the balance between time of staff to log behaviors during an active session and the types of information needed to aid in post-processing decisions.

Brief directions for staff were also included in the logs to provide reminders for key actions or events necessary for valid data (e.g., “Lights on”; “Check Flags”). Experimental staff also reported the number of trials attended, validity of each block of trials (data questionable, poor/no data, did not run), pauses (yes/no), and any additional notes to quantify child behavior during acquisition. After experiment completion, the staff marked overall behavioral data quality by experiment, including attention and affect, and identified the presence of other types of error (equipment, experimenter). Copies of the logs are available within the Acquisition Protocol documents (Barney et al., 2018; Borland et al., 2018; Sabatos-DeVito et al., 2018).

#### VT

All VT sessions were video recorded utilizing a standardized system (ceiling mounted Basler GigE IP camera with Pylon software interface to Noldus Media Recorder 3.0) and a second wall-mounted standalone side camera audio/video recording system (pre-existing at each site). The examiner at each site read standardized instructions describing the PCFP session. The camera operator sat in an adjacent room monitoring the Noldus and side camera recordings for QC and protocol compliance during the session. The camera operator informed the examiner of any compliance problems or deviations during the session.

#### EEG

All EEG sessions were videotaped, with video time locked to the NetStation recording. In our two-staff acquisition protocol, the experimenter monitored the acquisition computers, incoming EEG activity, and participant behavior via a real-time video embedded in the NetStation recording (Figure 2). The behavioral assistant, sitting next to the child, provided direction, prompts, and other supports to the child as manualized. The experimenter also coded child non-attention or other off-task behaviors via a keyboard response, which inserted a marker into the EPrime file and transferred to the NetStation EEG recording for file markup. At block breaks and end of experiment, the staff were presented (within the display) the number of attended trials.

#### ET

All ET sessions were videotaped and multiplexed onto a four-screen display that showed the participant, the Stimulus Presentation screen, and the ET Host screen, with the fourth screen left blank. The date and time was overlaid on top of this video and recording started before the child entered the room. In our two-staff setup, the experimenter monitored the stimulus presentation, tracking of the eye, and participant behavior via the four-screen display. The behavioral assistant, seated near the child, provided direction, prompts, and other supports to the child as manualized. The experimenter manually accepted each calibration point while the child was looking at it and could repeat points as necessary. The experimenter also had the ability to insert breaks or re-calibrations into the paradigms based on the data quality and the child’s needs using keyboard presses. Verbal re-directions, provided by the behavioral assistant to the child, were coded by the experimenter using keyboard presses. The use of these keyboard shortcuts was manualized in the protocol.

### Feasibility Study

The Feasibility phase included 51 participants (*n* = 26 ASD; *n* = 25 TD) aged 4–11 years. For Feasibility, we specifically addressed whether or not the methods and experiments could be successfully acquired for the participant groups. The sites were directed to each enroll 10 participants, five ASD and five TD. Sites were not directed to target enrollment by other characteristics due to the limited time window for this phase of the study. Across the Feasibility Study, we enrolled 73% male, 61% “older” (8–11 years sample), all with some verbal language.

Because feasibility of acquisition was a key outcome metric, we counterbalanced the method order (Table 1). Our initial biomarker battery included a one visit (or timepoint), two-day protocol with behavioral measures, EEG, and ET on both days. The PCFP with VT occurred in conjunction with the Autism Diagnostic Observation Schedule (day 1). EEG and ET occurred on both days 1 and 2.

Between days 1 and 2, the family took home the language environment analysis (LENA) system’s digital language processor (DLP) to record language use in the home. LENA is an automated system that analyzes recorded speech and other sounds in the natural home environment (Xu et al., 2009) and has been used to explore the language environment of children with ASD and TD (Warren et al., 2010).

For EEG, the experiments were divided into two sets with the method order randomized (Table 2). In terms of task ordering, the only experiment that was fixed was the EEG resting eyes open experiment, which occurred on both days in the first position. The projected time of the EEG experiments (from time of start of first experiment, to end of last experiment) for Set 1 was 12.5 min and for Set 2 was 13.5, not including breaks. During Feasibility, the mean actual run time was for Set 1 was 13.0 min (SD 6.8), and Set 2 was 15.0 min (SD 6.9).

For ET, the experiments also were divided across 2 days (see Table 2). Most paradigms were split into two blocks that were broken up by a Pupillary Light Reflex trial, with the exception of the longer videos (Spontaneous Social Orienting and Dynamic Naturalistic Scenes). The ET experiments were counterbalanced across four orders (e.g., Table 3, Order A) and experiments were interleaved to reduce fatigue and boredom. During Feasibility, the total projected time of the experiments (including setup, calibration, to end of the experiment set) over 2 days was 22 min; mean actual run time was 25.9 min (SD 3.3).

Note: Results from the Feasibility Study have not been published but were presented both at internal meetings with our advisory board and to the FNIH Biomarkers Consortium; based on investigator interest, some results were presented at scientific conferences and are available via the ABC-CT website.

## Main Study Acquisition

After review of results from the Feasibility Study, including review from NIH scientific and program officers, our external advisory board, and the FNIH Biomarkers Consortium, an overall decision was made that the battery was potentially burdensome for the child/family and that analytic interpretations could be confounded by the large number of derived result comparisons. To identify which measures were to be removed from the protocol, we first focused on feasibility using acquisition rates, protocol violation rates, and feedback from site coordinators. Second, we examined group discrimination (ASD versus TD), and reviewed our DVs for those that had *F* ≥ 1.9, which would reflect a power of 80%, and a potential significant result with our planned main study sample size. Third, we then examined redundancy in construct and DV between experiments. Fourth, we considered theoretical and practical barriers to eventual biomarker deployment in our target population in the context of clinical trials.

To this end, we made the following changes: (1) We discontinued use of the LENA system. LENA acquisition was poor in our Feasibility Study, with low return rates of the DLP (41% failure to return at day 2), and only 68% of recording sessions passed QC review. (2) We maintained the PCFP VT despite 91% of sessions reported as containing protocol deviations, with the majority reflecting failure to adhere to the standard room layout. We identified this as modifiable and revised the site initiation and training for the PCFP/VT. We also added a no-go criteria at the interim analysis for this paradigm. (3) We reduced the EEG acquisition to 1 day as site feedback identified high burden of netting participants twice within a timepoint. We also reduced the battery to four experiments (Table 2). While all experiments had good rates of usable data, emotion faces was removed because it had a lower acquisition rates (82%), did not discriminate groups (ASD versus TD: *F* = 1.3 for N170 amplitude to fear faces), and the potential for construct redundancy (e.g., early stage face processing) and DV redundancy (e.g., P1 and N170 ERP components) with the faces experiment. Although social/non-social dynamic had good acquisition rates (92%), we removed it from the battery as there were concerns with the appropriateness of content (nursery rhymes) for our age group and DV redundancy (e.g., power across the frequency spectrum) with the resting EEG experiment. (4) We maintained the acquisition of ET on both days but reduced the battery to five experiments (e.g., Table 3). As all nine experiments had acquisition rates > 94%, we focused on discrimination and redundancy to guide this removal decision. We eliminated the gap-overlap task because it did not discriminate groups. As the other tasks showed group discrimination, we rank ordered them based on effect size and retained SS, social interactive, and activity monitoring. PLR was maintained as a metric of basic visual system integrity. Visual search was maintained because it was acquired interleaved with SS and there was concern that construct validity would be disrupted by removing it. Dynamic naturalistic scenes and spontaneous social orienting performed well on all metrics but were removed due to concerns about the general use of the stimuli (e.g., copyright concerns for future dissemination and age appropriateness, respectively).

For each biomarker methodology in the Main Study, detailed acquisition protocols and manuals of operations were created to serve as the technical record, training manual, and protocol for acquisition (Naples et al., 2018; Sabatos-DeVito et al., 2018; Shic et al., 2018; Webb et al., 2018). These served as the primary training documents for the Site staff to guide data acquisition and addressed counterbalancing, experimental acquisition, equipment and setup, protocol when the child was present, site staff roles, and data logs.

### Data Storage and Security

Each site had their own IRB and HIPAA compliant local storage and backup systems for VT, EEG, and ET data. All clinical and (bio)marker data were entered into the Data Coordinating Center database RexDB informatics platform^2^ (Prometheus Inc.), including the transfer of the large VT, EEG, and ET data files. Data uploaded from the sites was done through this secure system. Access was limited to authorized personnel and monitored by the project management team and the DCC. Sites did not have access to the data of other sites; and only the DCC and DAAC had access to the full study data. QC review for correct stratification order was checked using grouping characteristics provided at screening (age group, diagnosis group, sex, and functioning). All review of participant data (VT, EEG, and ET files) was done blinded to participant (clinical and cognitive) characteristics except for site and date.

### Quality Control

The DAAC received all raw (bio)marker data files from the DCC, conducted QC checks on data acquisition, provided feedback to sites, and then implemented experiment-specific pipelines (which transformed the raw data VT, EEG, and ET to NDAR-compatible formats and then to the analytic pipelines for derived results). For QC, two versions were identified: Basic and Intensive. All files received basic review within 5 business days, which included evaluation of acquisition characteristics that were required for establishing validity (e.g., ET calibration; EEG net placement). For intensive review, videos were additionally checked for adherence to the protocol as well as less tangible qualities such as child–staff rapport. All files designated for intensive review were completed within 3 business days and written feedback was provided to the sites. For acquisition during the Feasibility Study, 100% of files received intensive QC review by the DAAC staff. For Main Study, the first 10 files from each site for the Main Study received intensive review. After these Main Study participants, a centralized list of participants was created with the data from every fifth child enrolled (by site and stratification group) being assigned to intensive review across modalities (that is, the same child received intensive review for VT, EEG, and ET and for all three timepoints) and the remaining participants received basic review. QC metrics were entered into the database for tracking and reporting. Quarterly reports were provided documenting percent of files that had been quality controlled, and percent valid. We have been able to maintain our feedback timeline for 94% of files.

Of note, acquisition QC is different than validity of derived results (i.e., a valid DV). The acquisition QC reflected adherence to the protocol and the ability of the participant to engage in the method acquisition for a minimum amount of time. As provision of either EEG *or* ET data was required to be maintained in the study, it was thus important to set a required minimum value of data that could quickly be accessed and communicated back to the sites to update recruitment goals. It was not deemed feasible to provide sites with information as to DV inclusion (i.e., did the participant have enough valid data to use in analysis) within the time frame needed to support recruitment. Moreover, balancing the need to have some amount of validly acquired data to proceed but also not requiring valid DVs, allowed us to compare characteristics of participants who might be included versus excluded if a specific biomarker was required for enrollment in a future clinical trial.

#### VT

Video tracking data quality was maintained by (1) confirmation of successful automated tracking of each child and (2) visual review of each recording (Sabatos-DeVito et al., 2018). Possible interference with tracking included objects of color similar to the child’s shirt, child not wearing the designated color for tracking, child’s shirt obscured from the overhead camera (e.g., hiding behind or under furniture, standing in a location not captured by the overhead camera), parent seating or interference, and furniture placement. Post session, acquisition was deemed valid if the child’s movement was successfully automatically processed. All files received review to confirm compliance with the PCFP protocol.

#### EEG

Post-session, acquisition QC was deemed valid if the participant had average or excellent EEG cap placement (both as reported by the site and validated via images of the participant), had completed 50% (out of 3 × 1 min blocks) of the EEG Resting State experiment (from the EEG logs), and if the EEG recording file was readable with the expected experimental markers (Santhosh et al., 2018). Additional factors were reviewed such as naming of the file, implementation of the counterbalance order, electrode impedances and signal quality, and protocol deviations. During intensive review, the full log was compared to the video recording and the EEG signal for congruence, electrode signal across the whole recording was reviewed, and the behavioral support was evaluated.

#### ET

Post-session, acquisition QC included confirmation that ET files were readable with the expected stimulus markers and that at least three of 16 blocks (20%) had data (Barney et al., 2018). Additional factors were reviewed such as file naming, valid on-screen looking percentage, calibration error, valid trials per paradigm, proper counterbalance order implementation, session duration, and appropriateness of keyboard shortcuts for recalibration and breaks. During intensive review, the full ET Run Log was reviewed alongside the video recording to ensure that the protocol was being followed and that appropriate behavioral supports were being utilized.

### Acquisition Results

All methods were attempted with all participants and valid acquisition of either ET or EEG at Time 1 was required to continue in the protocol. Given high rates of acquisition for the Feasibility Study, we focus on the Main Study interim results, which included 161 ASD and 64 TD participants enrolled between October 7, 2016 and December 1, 2017. In planning our interim report timeline, we pre-identified the date at which approximately 50% of the sample would have provided valid derived results for Time 1 and Time 2 based on a prespecified attrition rate (20%) and data loss rate (30%). (Note, as reported in the Supplementary Material, our attrition rate in the interim sample was only 2%.).

As identified in Table 4, acquisition rates at the interim analysis are based on inclusion in the study and provision of data that passed our QC criteria (section “Data Storage and Security”). Collapsing across Time 1 and Time 2, we had 100% valid acquisition for ET reflecting the low behavioral demands of the protocol and the rigor of the equipment hardware and software setup. VT valid acquisition was also high (96%) and the EEG session acquisition validity was 95–96%. We also tracked protocol deviations to identify when data were acquired in a non-standard manner but the deviations did not impact the ability to process the data using the analytic pipelines.

**TABLE 4:** Acquisition quality control rates for VT, ET, and EEG for Feasibility, and Main Study Time 1 and Time 2 at Interim analyses.

QC table	VT			ET			EEG		
			
	F	MS T1	MS T2	F	MS T1	MS T2	F	MS T1	MS T2
N	51	225	224	51	225	225	51	225	225
Acquire	51	225	224	51	225	225	51	222	222
Pass QC	50	216	215	50	225	225	50	216	215
%	98%	96%	96%	98%	100%	100%	98%	96%	95%
Protocol deviations	49 = 98%	40 = 18.5%	48 = 22.3%	5 = 10%	21 = 9.3%	22 = 9.8%	4 = 7.8%	35 = 16%	19 = 9%

*Key: F = Feasibility, MS = Main Study, T1 = Time 1; T2 = Time 2/ + 6 weeks.*

### Dependent Variable Specification

As part of the pre-specification of our Interim Analysis Plan, each method specified a primary experiment and primary and secondary DVs. Consideration focused on: Construct validity, that is, did the experiment elicit the intended processes? And group discrimination, that is, were there mean differences in the biomarker variables at Time 1 between the ASD and TD groups? The primary experiments/primary DVs included the (1) EEG resting eyes open experiment with slope of the power spectrum (over the whole head); (2) ERP ABC-CT faces with the N170 latency to the upright faces at the posterior right region of interest; (3) ET composite which included the average percent looking to heads for activity monitoring, social interactive, and SS; and (4) VT latency to approach the periphery. Primary variables for each of the experiments are listed in the header row for Tables 5, 6.

**TABLE 5:** Main Study Interim Time 1 VT and EEG experiments: percent of the children contributing valid data and test–retest reliability ICCs.

	VT PCFP, latency to approach periphery	EEG resting, slope	ERP faces, upright face N170 latency	ERP VEP, checkerboard P1 amplitude	ERP biomotion, biological motion N2 amplitude
**Percent of the children who provided a valid primary dependent variable value**					
Total	94%	91%	80%	81%	59%
ASD	94%	89%	74%	80%	55%
TD	92%	97%	92%	86%	69%
**Test–retest reliability (T1 to T2) ICC**					
Total	0.14	0.82	0.68	0.73	0.03
ASD	0.11	0.83	0.66	0.68	−0.09
TD	0.21	0.83	0.66	0.80	0.21

**TABLE 6:** Main Study Interim Time 1 ET experiments: percent of the children contributing valid data and test–retest reliability ICCs.

	ET composite, % Heads	Activity monitoring, % Heads	Social interactive, % Social	Static scenes, % Face	Biological motion, % Affective	Pupillary light reflex, latency to max constriction
**Percent of the children who provided a valid primary dependent variable value**						
Total	98%	100%	99%	100%	99%	96%
ASD	97%	100%	99%	100%	99%	96%
TD	100%	100%	100%	100%	100%	98%
**Test–retest reliability (T1 to T2) ICC**						
Total	0.83	0.85	0.54	0.53	0.32	0.73
ASD	0.79	0.79	0.35	0.54	0.36	0.69
TD	0.83	0.83	0.13	0.42	0.23	0.82

*Composite = activity monitoring, social interactive, static scenes. % = percentage of time spent attending to a specific region of interest within an image or condition.*

### Analysis Plan

One of the key principles of the Main Study Interim Analysis Plan was to ensure that all study processes were on track, potentially identifying issues that would result in changes to the protocols or recruitment strategies. As noted in our QC analytics, rates of valid acquisition across the three methods (VT, EEG, and ET) were high across the sites, highlighting the success of our development, training, and acquisition protocols. Second, and of importance to our final study goals, the interim analysis provided preliminary identification of the DV*s* that might have the best potential to serve as (bio)markers in clinical trials, both in terms of their core acquisition and psychometric properties and their utility for discrimination. Thus, our Interim analysis plan also focused on the rates of acquisition of our pre-specified primary and secondary DVs. That is, if a participant provided validly acquired data, we then examined the rates for which that raw data resulted in a valid dv value.

#### Biomarker (Dependent Variable) Acquisition

To be considered a valid biomarker, several key characteristics were deemed critical. First, the marker needed to demonstrate high acquisition rates across sites and across key demographic/clinical factors, including age, gender, and functional level. We proposed that an acquisition disparity of less than 20% between subgroups would suggest that a biomarker could be used broadly within a sample of children with ASD. Disparities of greater than 20% in acquisition rates and valid DV rates would suggest that the biomarker would not be appropriate for broad clinical trials, particularly as an inclusion requirement or primary outcome. As seen in Tables 5, 6, we provide the rates for our pre-specified primary DVs for each experiment, for our Time 1 Interim sample by group. Both ET (Table 6) and VT (Table 5) demonstrated high rates of valid abstraction of the primary variables; that is, data “loss” during post acquisition processing was low.

Electroencephalography showed significantly greater data loss when comparing acquisition rates to DV abstraction (compare Table 4 with Table 5). There were general concerns for abstraction rates of the primary variables for the ABC-CT (ERP) Biomotion Experiment, with overall lower rates of signal acquisition in both groups, making it problematic for use broadly. We also noted a significant decrease in valid DV rates within the ASD group with participants with IQ ≤ 70 (*n* = 17) for two of the ERP Experiments (ABC-CT Faces 35%, Biomotion 29%) and all experiments had a > -20% difference in inclusion rate for between ASD IQ > 70 compared to ASD IQ ≤ 70. ERP visual experiments, in general, require fixed visual attention to the screen and thus are “harder” for participants with attention deficits. While it is possible that alternate protocols would improve rates of attended/artifact free trials, clinical trial protocols would need to consider the relation between child characteristics and provision of data.

#### Biomarker (Dependent Variable) Distribution

Our second set of validity characteristics focused on the statistical properties of the candidate biomarker variables. We proposed that the biomarker values must demonstrate appropriate distributional properties, such as absence of severe non-normality, skew (values < 2, with checking for values≠ 0), kurtosis (values < 3, with checking for values≠ 0), floor/ceiling effects, and zero inflation. Floor and ceiling effects may suggest that the variable fails to cover the range of the construct; while zero inflation may suggest that the experiment manipulation failed to evoke the behavior of interest. Note that consideration of the distributional properties was done in parallel with confirmation of construct validity. For example, one goal of a potential stratification biomarker might be to identify a process that differs in the TD and ASD group. In this case, the variable of interest may show a distribution with substantial regions of non-overlap or a different probability concentration; correspondingly, the presence of distributional issues in the ASD group but not the TD group could represent an important signal and hence would not be disqualifying. Variables that exhibit multi-modality may also indicate a natural separation into subgroups. Further, potential outliers may be indicators of a separate underlying (pathophysiological) process.

At interim, all of our EEG and ET variables demonstrated adequate distribution. However, for the VT analyses and as discussed in Murias et al. (2019), the data for PCFP *latency to approach periphery* showed a significant number of participants had a valid minimum (0 s) or maximum (360 s) value, reflecting that some participants began the PCFP in the periphery region, while others never moved into the periphery region of the room, preferring to play in the activity regions (table or center) or near the caregiver. In Figure 1A, the child moved between caregiver, central toys and table; while in Figure 1B, the child remained only near the central toys. It is important to note that the VT itself worked reliably; instead it is the interaction with the construct of interest (child approach behaviors during the PCFP) that demonstrated limitations. Thus, because there were concerns both about the distribution of this variable and the construct as operationalized, it was deemed to have failed the go/no-go criteria.

#### Biomarker (Dependent Variable) Test–Retest Reliability

Third, the biomarker must show moderate test-retest reliability in the TD control group. This was based on an expectation of no (meaningful development or environment/treatment related) change over a 6-week (Time 1 to Time 2) period in the TD group. While we did analyze test–retest reliability in the ASD group, we did not pre-specify a required value for evaluation of the biomarker as we did not require that participants maintain treatment stability after enrollment into the study. For example, we would expect that participants with ASD might experience changes in treatment service availability (i.e., therapist vacation or start of school year) or potential need for medication adjustment.

To assess test–retest in both groups, we used intra-class correlations (ICCs) using mixed models with a random score/fixed rater structure and the absolute agreement metric. This provides a version of the correlation accounting for potential mean drift. For test–retest reliability, we pre-specified that, at Interim and for the TD group, excellent rates of test–retest would be represented by ICC of > 0.75, with adequate as 0.50–0.74, and concerns at <0.50. As shown in Table 5, the VT variable had distributional concerns and showed poor test–retest reliability. For EEG, values were adequate for three of the four primary variables. The Biomotion N2 amplitude to biological motion proved concerning. For the ET composite variable (which combines the primary DV from the activity monitoring, social interactive, and SS), the ICC value was excellent, and performed better than any of the individual variables. It also should be noted, that contrary to prediction, some of the ET experiments and primary variables had lower ICC values in the TD than ASD group (although they were not statistically compared). This may reflect the “artificial” nature of the social stimuli and their development as experiments that address autism specific social *disability*.

As part of the Main Study Analysis, a priority will be understanding the variables that impact test–retest reliability. Specifically, we will address how the interaction between the diagnostic groups and other demographic characteristics (e.g., age, sex, functioning) impacts test–retest as this may inform subgroups for which measures may be more appropriate in clinical trials. Second, we will identify the extent to which clinical change in symptoms or in (behavioral or medication) interventions may impact the DVs of interest. Differences in a change versus no change group, even at a global level may inform us of which biomarkers are more malleable. Third, we will examine the extent to which measurement acquisition variability influences test-retest values. To address this, we will examine (1) variables that may be modifiable within the protocol such as time of day of assessment; (2) variables that may be addressed through post-acquisition processing such as within-child matching of percent valid-included data; and (3) variables that may be difficult to address in a clinical trial such as changes in child non-compliance. Of importance for clinical trials, is the extent to which the primary DVs are “fragile” in ways that can neither be addressed in the protocol nor corrected for (or normalized) in interpretation of the values, making it difficult to identify change related to treatment effects.

## Limitations

Because acquisition metrics are central to understanding how VT, EEG, and ET biomarkers might work in a future trial, it was equally important to understand who could *and* could not provide valid data, and thus we fixed aspects of the protocol and limited site variability that may have disadvantaged individual participant performance. For example, we did not allow for multiple testing attempts or alteration of protocol order. For some children with ASD, a longer phase of exposures to the equipment or environment may facilitate comfort and compliance. As well, task order was fixed such that an individual experimenter could not reduce the burden of the number, length, or types of paradigms for a child. For the EEG battery, sensory sensitivities (to the net) and focused visual attention might limit the length of time the child could engage with the equipment or the task. Given the specificity of treatment targets, we might expect that a smaller set of methods and experiments would be employed in a clinical trial, reducing the burden on the participant and the experimental teams. We suggest that similar types of QC metrics be applied, however, to ensure that any variability in performance is not due to site implementation.

Second, while we allowed sites individual flexibility in preparing the participant for tasks and using the individual’s support tools, we did limit some types of engagement around the protocol. For example, language describing the tasks and stimuli was prescribed and we did not allow for modifications to the environment. We also did not allow for modifications such as reducing the number of measures per visit or allowing multiple visit attempts. It is possible alternative individual modifications could have been considered that would have benefited acquisition while preserving the integrity of the task (Webb et al., 2015). While some environmental changes (like ambient lighting) are known to impact performance on certain measures (e.g., pupillary light response), there are others where the impact is less clear. The difficulty of teasing apart the impact of individual modifications and the resulting performance is that we might expect that children that are the most impaired are not only most likely to need modifications to the protocol but also the most likely to have outlier or atypical responses. Thus, differentiating whether or not the responses are related to the individual’s phenotype or to the modifications will require additional study.

Third, all ABC-CT sites had significant experience collecting behavioral, EEG, and ET data for research purposes and all site PIs had > 10 years of experience with the specific EEG hardware and software employed in this protocol. Thus, sites entered the study with a demonstrated track record in acquisition, analytics, and dissemination. In addition to the ABC-CT, two other large efforts are addressing the issue of “translational neuroimaging” with the goal of improving clinical trial measurement; both the EU-AIMS LEAP (e.g., Loth et al., 2017) and Janssen Autism Knowledge Engine (JAKE) study (e.g., Ness et al., 2017). In contrast to our protocol, EU-AIMS LEAP has greater site variability in equipment and populations included, while JAKE was specifically designed to allow acquisition to occur in clinical environments. The contrast of results from these will provide insight into standardization requirements. Regardless, with novice sites, it would be expected that a longer training or feasibility phase might be needed to address experience. The use of formalized QC feedback, delivered with 3–5 days of acquisition, also supports early identification of protocol drift or need for re-training.

Fourth, within the scope of this report, we have focused on our acquisition protocol and acquisition QC metrics. All three methods detailed also have extensive post-acquisition processing pipelines wherein the raw data are transformed into analyzable DVs. These protocols will also be detailed in manuals that will be available to the scientific community. The reliability of our results is not only contingent on acquisition procedures but also the definitions of artifact and signal that are implemented in post-acquisition data pipelines. These details will be included in our empirical papers and discussed in relation to their impact on our conclusions.

## Conclusion

Based on the preliminary acquisition metrics, experiments that utilize VT, EEG, and ET in a sample of children with ASD can be acquired across multiple academic laboratories utilizing a well specified, manualized standard training and acquisition protocol with significant success. Our ABC-CT protocol for successful acquisition includes development and utilization of standardized equipment and experiments; on-site training and consistent, regular contact between acquisition leads and experimenters; and manualized QC and feedback. Our Interim Analyses stressed the importance of validity of acquisition, including equivalent functioning across site and participant characteristics, distributional properties, and test–retest validity as these are critical in evaluating the suitability of a biomarker for use in a clinical trial context. Final analyses with the full Main Study sample will offer the opportunity to explore discrimination, factors that impact test–retest reliability, clinical and behavioral correlates, supervised stratification, multivariate biotypes, and naturalistic illness trajectories. Ultimately, preliminary clinical trials will be required to validate candidate biomarkers for context of use and acquisition metrics (FDA-NIH Biomarker Working Group, 2016). Overall, our ABC-CT protocol demonstrates a successful framework for the analytic validation of potential (bio)markers for use in autism and other neurodevelopmental disorders. The next step will be to move to qualification and utilization (Institute of Medicine (US) Committee on Qualification of Biomarkers and Surrogate Endpoints in Chronic Disease, 2010).

## Data Availability Statement

The ABC-CT data can be found in the National Database for Autism Research https://ndar.nih.gov/, collection ID #2288.

## Ethics Statement

This study was carried out in accordance with the recommendations of “Yale University Institutional Review Board” with written informed consent from parents of all child participants. All parents gave written informed consent in accordance with the Declaration of Helsinki. The protocol was approved by the “Yale University Human Subjects Committee.”

## Author Contributions

All named authors made substantial contributions to the conception or design of the work and read and provided approval for publication of the content. SW, FS, MM, AN, EB, MK, MS-D, MS, and DS contributed to the drafting of the work. SW, FS, MM, AN, EB, MK, MS-D, MK, AL, MS, DS, RB, KC, GD, and JM provided critical revisions related to the important intellectual content.

## Conflict of Interest

EB was employed at Seattle Children’s Research Institute at the time of the drafting of this manuscript; she is currently of Cogstate (www.cogstate.com). RB was employed at the University of Washington at the time of the submission of this manuscript; he is currently employed by Apple. MK was at Seattle Children’s Research Institute at the time of the drafting of this manuscript; she is currently at University of Virginia. MM was at Duke University at the time of the drafting of this manuscript; he is currently at Northwestern University. GD is on the Scientific Advisory Boards of Janssen Research and Development, Akili, Inc., LabCorp, Inc., and Roche Pharmaceutical Company, a consultant for Apple, Inc., Gerson Lehrman Group, and Axial Ventures, has received grant funding from Janssen Research and Development, is CEO of DASIO, LLC, which focuses on digital phenotyping tools, and receives book royalties from Guilford Press, Springer, and Oxford University Press. JM has received funding from Janssen Research and Development and receives book Royalties from Guilford, Springer, and Lambert Press. FS consults for Roche Pharmaceutical Company and Janssen Research and Development. The remaining authors declare that the research was conducted in the absence of any commercial or financial relationships that could be construed as a potential conflict of interest.

## Footnotes

1 www.asdbiomarkers.org

2 https://www.rexdb.org
