# Supplementary material for: Biomarker Acquisition and Quality Control for Multi-Site Studies: The Autism Biomarkers Consortium for Clinical Trials
Source: Front Integr Neurosci. 2020 Feb 7;13:71. doi: 10.3389/fnint.2019.00071 (PMC7020808; doi:10.3389/fnint.2019.00071)
Supplement: Supplementary file 1 [file Table_1.docx]

**S1 Methods**

**S1.1 Interim Sample Characteristics**

Of the 250 participants who enrolled and were included in the ABC-CT Time 1 Interim Sample, 4 discontinued before Time 2 (ASD=3; TD=1) and 1 additional youth discontinued at Time 3 (ASD=1; TD=0).

In Supplemental Materials Table 1, we provide sample characteristics from the 250 participants included in our interim analysis who completed Time 1 by 12/1/2017. Sites contributed between 39 to 49 participants to the interim sample, with no differences in percent ASD, male, race, cognitive ability ≤70 (χ^2^(4)<4.0, ps= ns) nor age, the Differential Abilities Scale 2 (Elliott et al., 2006) Full IQ, Verbal IQ, Nonverbal IQ; ADOS Calibrated Severity Score (Gotham, Pickles, & Lord, 2009), SRS Total (Constantino, 2013), or Vineland Adaptive Behavior Scales Socialization Score (VABS-3; Sparrow et al., 2016) (F<2.3, p>.06). Sites did differ in Hispanic ethnicity (χ^4^(4)= 13.4, p=.01), with ranges in Hispanic participants between 2% to 27% by site.

**Supplemental Materials Table 1. Interim Sample Characteristics at Time 1 in the ABC-CT Main Study (Bernier et al., 2018; Faja et al., 2018).**

| **N=225** | **TD**  **n (%)** | **ASD**  **n (%)** | **Group comparison** |
| --- | --- | --- | --- |
|  | 64 | 161 |  |
| Male (%) | 42 (66%) | 131 (81%) | χ^2^(1)=5.6, *p*=.01 |
| Race= Caucasian  Ethnicity= Hispanic | 50 (78%)  6 (9%) | 113 (70%)  31 (19%) | χ^2^(1)=1.5, *p*=.21  χ^2^(1)=3.6, *p*=.06 |
| Older (>8.5 years) | 35 (55%) | 80 (49%) | χ^2^(1)=0.5, *p*=.47 |
| Cognitive Level (best IQ ≤ 70) | na | 17 |  |
|  | **TD**  **Mean (sd)** | **ASD**  **Mean (sd)** | **Group comparison** |
| Age in Years Mean (sd) | 8.7 (1.8) | 8.7 (1.6) | *F*(1,222)=0.0, *p*=.89 |
| Full IQ Mean (sd) | 114.6 (13.5) | 95.7 (18.9) | *F*(1,222)=53.3, *p*<.01 |
| Verbal IQ Mean (sd) | 116.3 (12.6) | 94.3 (21.0) | *F*(1,222)=61.2, *p*<.01 |
| Nonverbal IQ Mean (sd) | 111.6 (96.7) | 96.7 (17.4) | *F*(1,222)=36.1, *p*<.01 |
| ADOS Calibrated Severity Score  ADOS Social Affect CSS | 1.4 (.7)  1.8 (1.2) | 7.8 (1.8)  7.6 (1.8) | *F*(1,223)=751.0, p<.001  *F*(1,223)=558.3, p<.001 |
| SRS-2 Total Scale Score | 12.0 (87) | 83.7 (34.2) | *F*(1,222)=252.3, *p*<.01 |
| VABS-3 Socialization Score | 106.5 (7.7) | 70.9 (16.7) | *F*(1,222)=262.8, *p*<.001 |

## S1.2 References

Bernier, R., Dawson, G., and the Autism Biomarkers Consortium for Clinical Trials (2018, April). Demographics and Clinical phenotype. ABC-CT Annual In-Person Meeting, Boston, MA.

Faja, S., Bernier, R., Dawson, G., Chawarska, K., Jeste, S., Nelson, C.A., Webb, S.J., Shic, F., Naples, A., Sugar, C., Murias, M., Dziura, J., Brandt, C., McPartland, J. (May 2018). The Autism Biomarkers Consortium for Clinical Trials: Clinical Characteristics and Interim Evaluation of Clinical Measures Commonly Used in Clinical Trials. Oral Presentation 26826, 177.002. International Society for Autism Research. Rotterdam, Netherlands. Recorded presentation available at: https://insar.confex.com/insar/2018/webprogram/Session3930.html

Constantino, J. N. (2013). *Social responsiveness scale* (pp. 2919-2929). Springer New York.

Elliott, C. D., Salerno, J. D., Dumont, R., & Willis, J. O. (2006). Differential ability scales. Second edition.

Gotham, K., Pickles, A., & Lord, C. (2009). Standardizing ADOS scores for a measure of severity in autism spectrum disorders. *Journal of autism and developmental disorders*, *39*(5), 693-705.

McPartland, J., Webb, S.J., Shic, F., Naples, A., Sugar, C., Murias, M., Dziura, J., Brandt, C., Bernier, R., Chawarska, K., Dawson, G., Faja, S., Jeste, S., & Nelson, C. A. (2018, May). The Autism Biomarkers Consortium for Clinical Trials: Study Design and Progress to Interim Analysis, Oral Presentation 26826. International Society for Autism Research. Rotterdam, Netherlands. Recorded presentation available at: https://insar.confex.com/insar/2018/webprogram/Paper26826.html

Sparrow, S. S., Cicchetti, D. V., & Saulnier, C. A. (2016). Vineland adaptive behavior scales, (Vineland-3). *Antonio: Psychological Corporation*.
